# Characterization and pathogenicity of fowl adenovirus serotype 4 isolated from eastern China

**DOI:** 10.1186/s12917-019-2092-5

**Published:** 2019-10-28

**Authors:** Kai Wang, Haiwei Sun, Yunzhang Li, Zhiwei Yang, Jianqiang Ye, Hongjun Chen

**Affiliations:** 1 Shanghai Veterinary Research Institute, CAAS, Shanghai, 200241 China; 2 College of Veterinary Medicine, Yangzhou University, Yangzhou, 225009 China

**Keywords:** Fowl adenovirus serotype 4, Hydropericardium syndrome, Inclusion body hepatitis, Pathogenesis

## Abstract

**Background:**

Fowl adenovirus outbreaks have occurred in China since June 2015. This virus is an emerging infectious disease that causes hydropericardium syndrome and inclusion body hepatitis (HPS-IBH), resulting in significant economic loss to poultry farmers. Five fowl adenovirus (FAdV) strains (HN, AQ, AH726, JS07 and AH712) were isolated from Jiangsu and Anhui provinces.

**Results:**

Phylogenetic analysis revealed that the five isolates belonged to species C fowl adenovirus serotype 4. An 11 amino-acid deletion in ORF29, relative to an older viral isolate, JSJ13, was observed for all five strains described here. In chicken experiments, 80–100% birds died after intramuscular inoculation and displayed lesions characteristic of HPS-IBH. The viral DNA copies were further detected by *hexon*-probe based real-time polymerase chain reaction (PCR) in the chicken samples. The viral loads and cytokine profiles were recorded in all the organs after infections. Despite minor genetic differences, the 5 strains displayed significantly different tissue tropisms and cytokine profiles.

**Conclusions:**

Our data enhance the current understanding some of the factors involved in the pathogenicity and genetic diversity of the FAdV serotype 4 (FAdV-4) in China. Our work provides theoretical support for the prevention and control of HPS-IBH in chickens.

## Background

Fowl aviadenoviruses (FAdVs) are classified into five species (A-E) and 12 serotypes (FAdV-1 to 8a and -8b to 11) [1, 2]. FAdVs infects mostly 3–6-weeks-old broilers [3, 4], and is readily transmitted both horizontally by the fecal-oral route and vertically by embryonated eggs [5, 6]. Serotype 4 Fowl adenovirus (FAdV-4) belongs to group I adenoviruses in the genus Aviadenovirus, also known as ‘Angara Disease’, was first reported in Angara Goth, Pakistan in 1987. FAdV-4 causes Hydropericardium syndrome (HPS), Inclusion body hepatitis (IBH), respiratory tract disease, and/or gizzard erosion in chickens [7–9].

Since mid-2015, outbreaks of novel genotype FAdV-4 infections in China and Korea have caused great economic losses in the poultry industry, leading to high mortality, characterized by congestive kidney, pericardial effusion, hyperemic and enlarged liver with petechial hemorrhages and punctiform areas in broilers [10], commercial chickens [11], and ducks [12].

Recent Chinese FAdV-4 isolates have significant deletions in their genome compared with FAdV serotype 4 strains from other countries [13, 14]. However, the molecular mechanisms underlying the infection and pathogenesis of FAdV-4 remain unclear.

In the present study, several strains of FAdV-4 were isolated from Eastern China, their genomes were sequenced, and virulence assays were conducted in chickens.

## Results

### Identification and characterization of FAdV-4 variant isolates

**Fig. 1:**
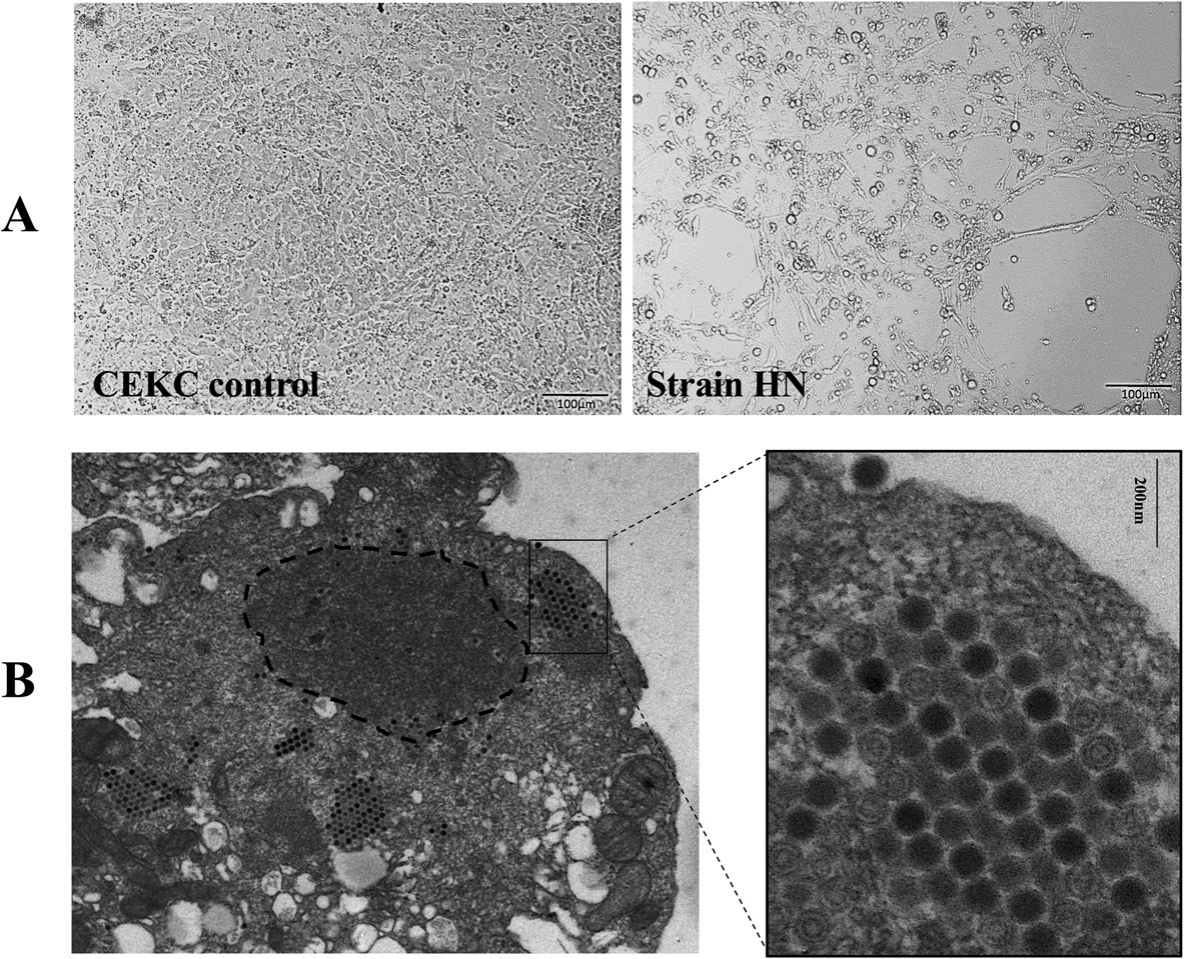
Cytopathogenic effects of FAdV-4 viruses in CEK cells. **a** The strain HN purified virus was passaged in 7 days - old SPF chicken embryonated eggs three times and then purified in CEK cells by plaque assay when typical CPE formation (bar = 100 μm). **b** Electron microscope of strain HN. The virus particles were observed as regular hexahedrons. The diameter was approximately 80–100 nm, which were shown as crystal arrangement in the cytoplasm of CEK cells (bar = 200 nm)

Chicken liver samples containing lesions indicative of inclusion body hepatitis-pericarditis syndrome were inoculated into SPF eggs. The embryos died after 3–7 days, showing classical lesions of fowl adenovirus infection with dysplasia, surface flushing, and bleeding. The livers of the dead embryonated eggs contained yellow-brown blood and necrotic spots. Allantoic fluids were collected and used for total viral DNA extraction, followed by PCR amplification of a conserved region of the *hexon* gene. Five viral isolates were identified as FAdVs and designated as HN, AQ, JS07, AH712, and AH726, respectively (the isolation of JS07 has been previously described by Wang et al., 2016 [15]. The samples were passaged in 7 day- old SPF chicken embryonated eggs and then purified in primary chicken embryo kidney (CEK) cells by plaque assay after typical CPE formation occurred (Fig. 1a). Virus titers in the infected embryos and CEK cells were 10^8^ to 10^8.5^ TCID_50_ / ml. Viral particles were observed to be regular hexahedrons using electron microscopy. The diameter of the viral particle was approximately 80–100 nm, which appeared as crystals arranged in the cytoplasm of CEK cells consistent with the characteristics of fowl adenovirus (Fig. 1b).

### Analysis of complete sequences of FAdV-4 isolates

**Fig. 2:**
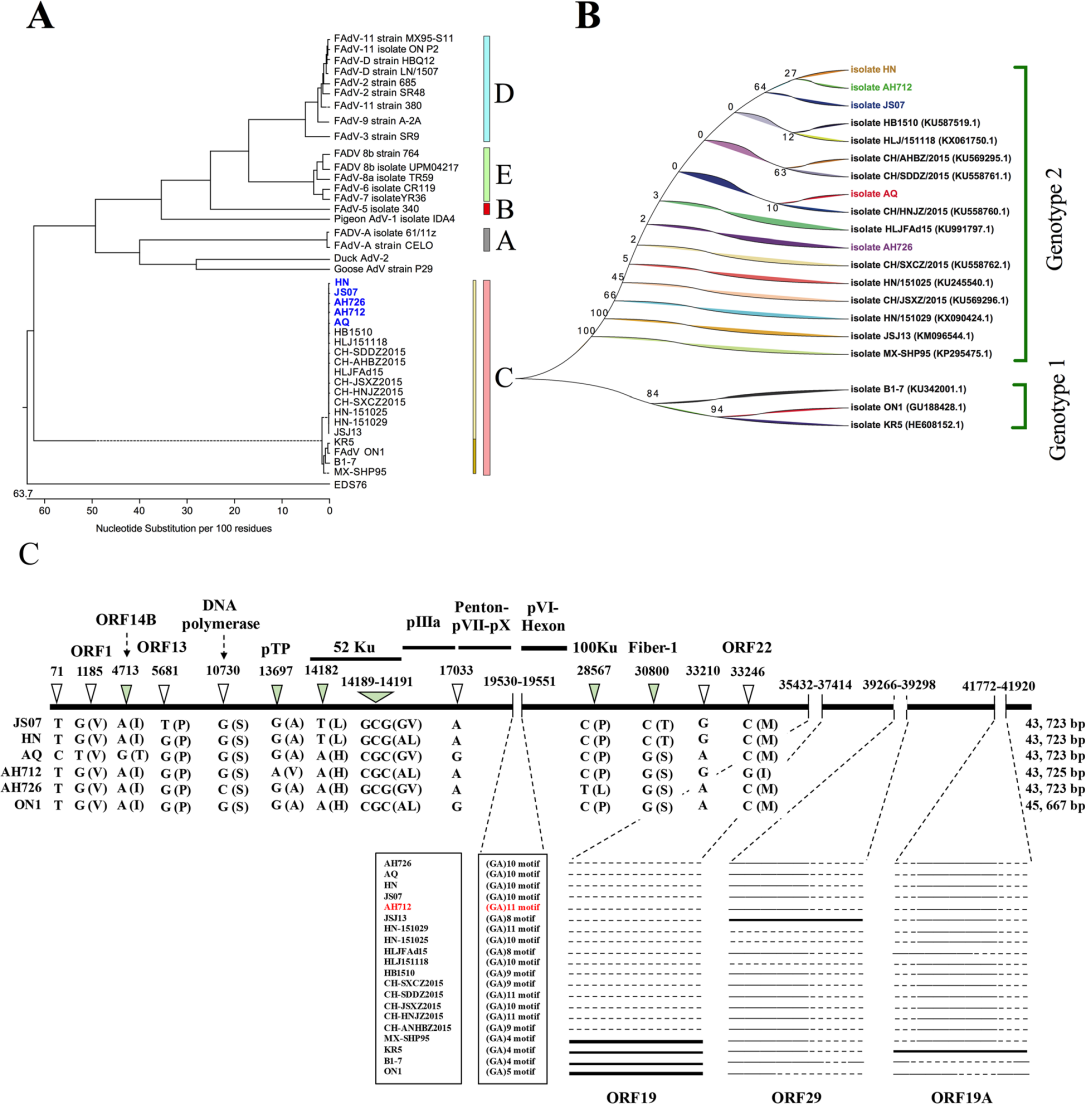
Phylogenic analysis of FAdV-4 isolates. **a** By phylogenic analysis based on the genomic sequences with ClustalW method, the 5 strains belong to species C FAdV serotype 4. **b** The FAdV-4 strains were divided into two genotypes. All the recent virulent strains fell into genotype 2, whereas three non-pathogenic strains, B1–7, ON1 and KR5 belong to genotype 1. **c** Comparison of the genomic sequences showed the mutations or deletions might be related to virulence. The brakets represent amino acids

To investigate the molecular pathogenicity of the isolates, the viral genomes were first sequenced. The genome of isolate AH712 was 43,725 base pairs (bp) in length, and the other four strains were 43,723 bp. The whole genome nucleotide sequences of the isolates were deposited in GenBank (Table 1). The strains belong to species C FAdV serotype 4 (Fig. 2a). Compared with ON1 (GU188428), a natural deletion of 1966 bp was observed at the position of nt 35,425 based on ON1’s genome. This deletion includes two open reading frames, ORF19 and ORF29 (Fig. 2c), which were also present in the recent Chinese variants [16]. The 5 strains are similar to the recently reported highly virulent HLJ/15118 strain, and the non-pathogenic strains clustered into a subgroup. Based on the alignment of the genome sequences of all FAdV-4 strains by the ClustalW method, the strains were divided into two genotypes. All the recent virulent strains were located in the genotype 2 of FAdV-4 (Fig. 2b), whereas three non-pathogenic strains, B1–7, ON1 and KR5 belong to genotype 1. Compared with the Chinese strain JSJ13 isolated in 2013, there were 33 nt deletions in the ORF29 sequence of the five strains. Compared with the classical non-pathogenic strain ON1, there were different levels of GAGA motif repeats in the isolates at 19530–19551 nt (Fig. 2c). The mutations were dispersed throughout the genes encoding the ORF14A, pTP, 52 K, and 100 K proteins (Fig. 2c). Comparison of variable amino acid sequences from Fiber-2 (FAdV surface Fiber protein 2) among HPS and non-HPS isolates are also shown in (Additional file 1: Table S3).

**Table 1:** The data of compete sequences of FAdV-4 isolates in unvaccinated chickens

Strain	Location	Chicken type	Collection date	Size (kb)	Accession Number	Titers (TCID_50_/mL)
JS07	Jiangsu	Broiler	2015.11.2	43,723	KY436519	2.32 × 10^7^
AQ	Anhui	San-Huang Layer	2016.6.28	43,723	KY436520	5.00 × 10^8^
HN	Anhui	San-Huang Layer	2016.6.28	43,723	KY379035	2.81 × 10^8^
AH712	Anhui	817 broiler	2016.7.12	43,725	KY436522	1.08 × 10^8^
AH726	Anhui	San-Huang Layer	2016.7.26	43,723	KY436521	1.58 × 10^8^

### Quantification of FAdV-4 virus particles by TaqMan probe real-time PCR

We developed a TaqMan probe fluorescence quantitative polymerase chain reaction (qPCR) method to rapidly and accurately detect and quantify FAdV-4. We used a pGEM-T easy-hexon plasmid to generate the FAdV-4 DNA standard curve. The standard linear equation is y = − 3.52x + 35.98 (*R*^*2*^ = 0.998). The lowest limit of detection was 22.8 copies/μl, which is 100 times higher than the conventional PCR method. The method laid the foundation for further detection of FAdV-4 isolate in vitro and in vivo, as well as a tool for rapid clinical diagnosis of the disease.

### Pathogenicity of recent strains in SPF chickens

**Fig. 3:**
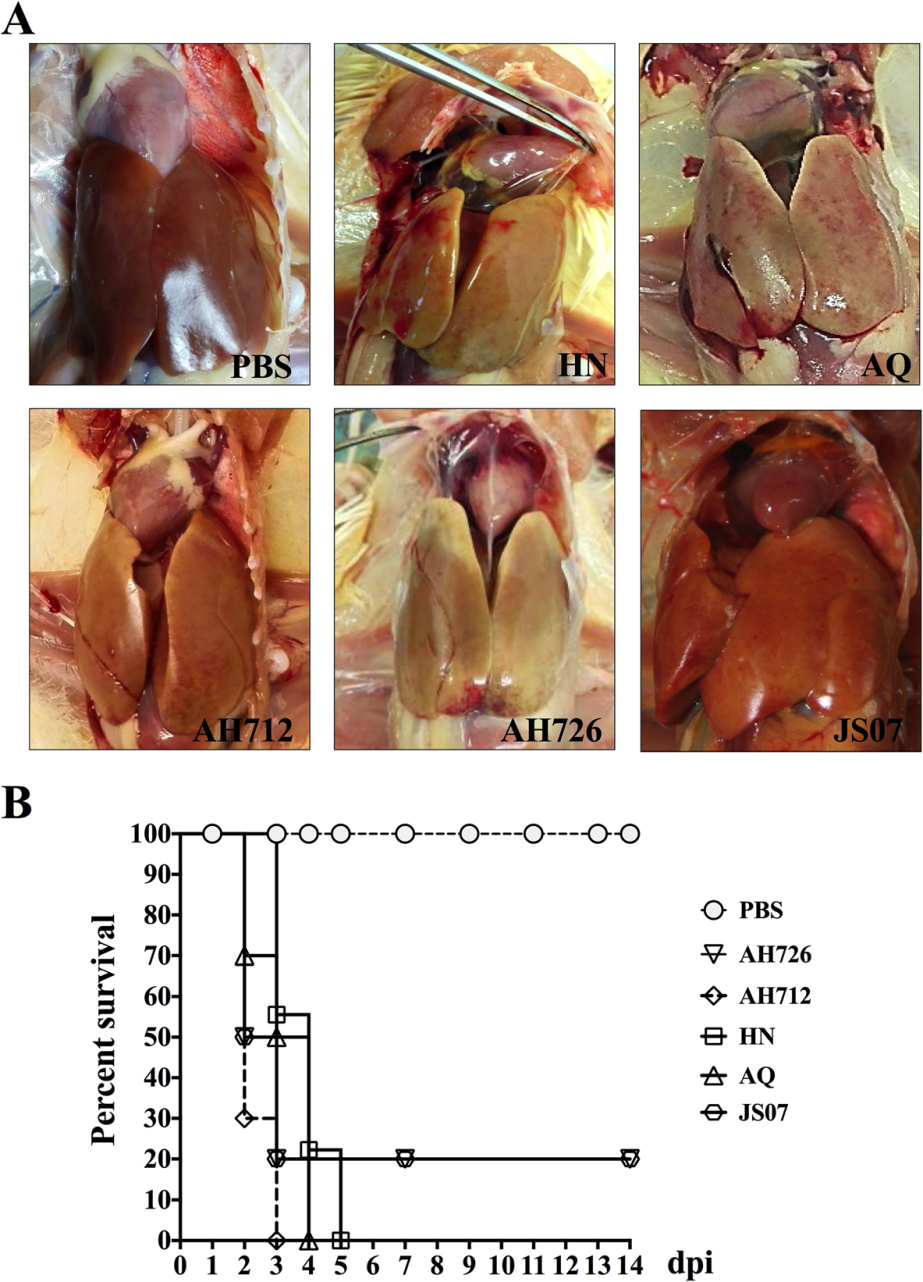
Pathogenicity of the recent strains in 3-week-old SPF chickens. **a** Gross lesions of FAdV-4 strains. All dead chickens in the infected groups showed severe HPS-IBH syndrome. **b** The survival rate of the isolates

**Fig. 4:**
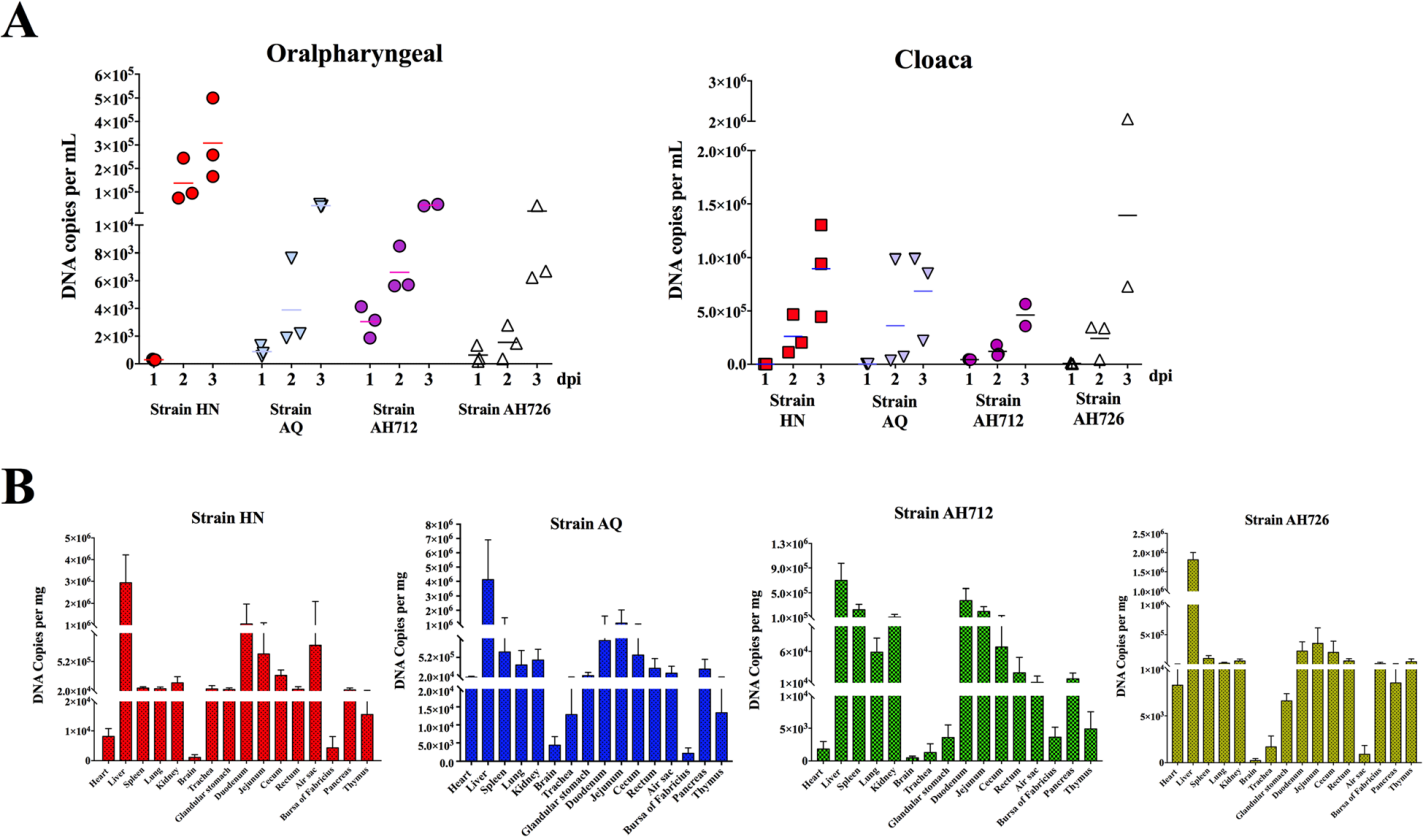
Viral genome copies in swab samples and infected tissues. **a** Viral loads were detected in the oralpharyngeal and cloacal swabs of infected chickens by TaqMan probe real-time PCR method based on *hexon* gene. **b** Viral genome copy numbers in heart, liver, spleen, lung, brain, trachea, glandular stomach, duodenum, jejunum, cecum, rectum, air sac, bursa of Fabricius, pancreas and thymus samples of strain HN, AQ, AH712 and AH726 challenge groups were determined and summarized

**Fig. 5:**
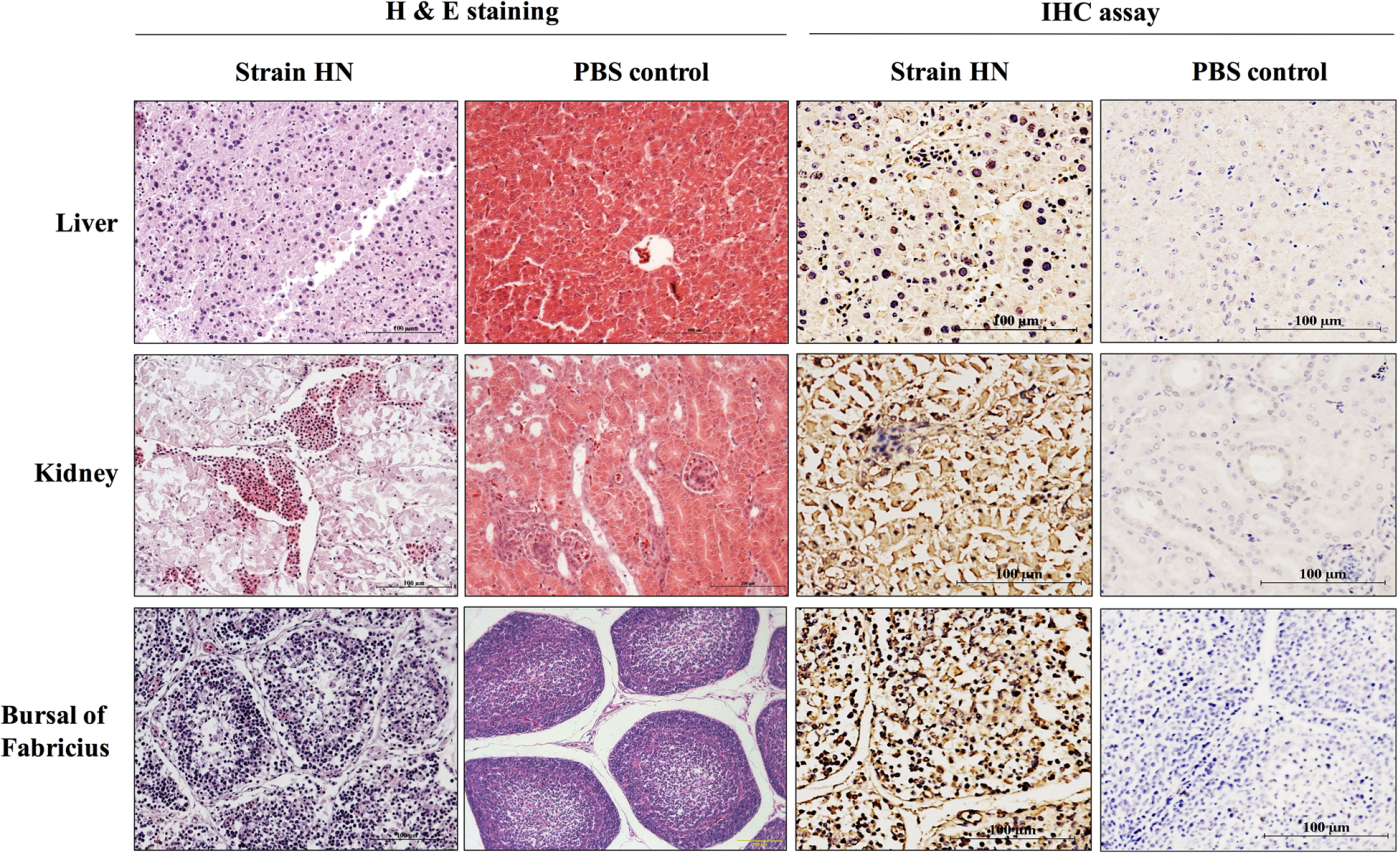
H & E staining and immunohistochemical (IHC) observation. The slices of strain HN - infected liver, kidney, and the bursa of Fabricius were observed by H & E staining and reacted with mAb 1B4 against Hexon by IHC assay. Magnification is × 20

All dead chickens in the infected groups showed severe HPS and IBH (Fig. 3a), with yellow and hemorrhagic livers and pericardial effusion (Fig. 3a). Tissue samples were collected and stored at − 80 °C for titration or in 10% neutral formalin for Hematoxylin & Eeosin (H & E) staining. In the infection experiment, the lethality rates of HN, AQ, and AH712 were 100%, and the lethality rates of JS07 and AH726 were 80% (Fig. 3b). Viral genome copy numbers in heart, liver, spleen, lung, brain, trachea, glandular stomach, duodenum, jejunum, cecum, rectum, air sac, bursa of Fabricius, pancreas, and thymus samples were determined using TaqMan probe qPCR and are summarized in Fig. 4. High viral loads were detected in the oralpharyngeal and cloacal swabs of infected chickens. All the isolates caused symptoms consistent with acute fowl adenovirus infection within 3 dpc. At 3dpc, the mean titers were 3 × 10^5^ DNA copies/ml in oral swabs and 1 × 10^6^ DNA copies/ml in cloaca swabs from the HN strain challenge group (Fig. 4a). High viral titers were observed in most organs, with livers having the highest viral loads, followed by the duodenum, jejunum, caecum, rectum, and finally the brain (Fig. 4b). No viral DNA was detected in chickens before inoculation or in mock-infected chickens. Infected chicken livers presented with typical basophilic inclusions, a large number of inflammatory cells infiltrated into the kidneys slices, and the infected bursa of Fabricius had structural disorders and reduced numbers of lymphocytes. The liver, kidney and bursa of Fabricius reacted with mAb 1B4 against Hexon, further indicating that presence of virus in these tissues (Fig. 5).

### High expression of cytokine genes during acute infection

**Fig. 6:**
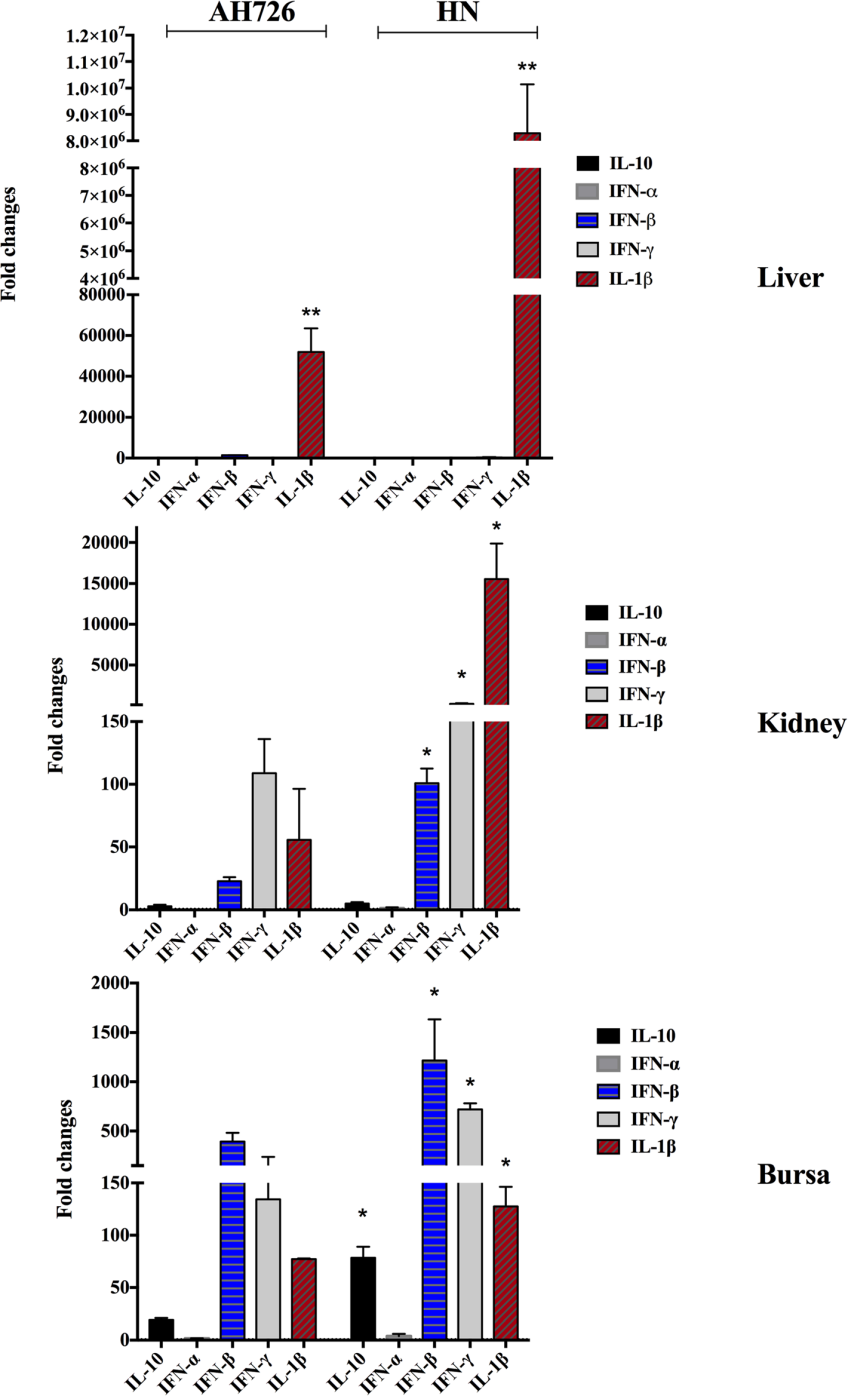
The changes of cytokines in the infected tissues. Expression of mRNA levels of cytokine genes after I. M inoculation of chickens with strains AH726 and HN were measured compared to the data of the mock as control. The fold changes on the mRNA transcriptional levels of *IL-1β, IFN-α, IFN-β*, *IFN-γ,* and *IL-10* were illustrated and calculated with the 2^-ΔΔCt^ method. Note: statistical significance as *p* < 0.05 (*) or *p* < 0.01 (**)

Based on the calculated lethality values, strains HN and AH726 were selected for further analyses. Cytokine gene expression following infection was measured in the liver, kidney and bursa of Fabricius. The expression levels of *IL-1β, IFN-α, IFN-β*, *IFN-γ,* and *IL-10* mRNAs were shown in Fig. 6. Compared to the PBS control group, there was a statistically significant increase in *IL-1β*, *IFN-β*, and *IFN-γ* transcriptional levels of the kidney and bursa of Fabricius samples in the infected chickens at 3 d.p.i (*p* < 0.01). However, in liver only *IL-1β* mRNA level was significantly higher in the infected group at 3 d.p.i (*p* < 0.01). Compared to the high virulence of strain HN with 100% mortality, the *IL-1β, IFN-α, IFN-β, IFN-γ,* and *IL-10* mRNA levels in bursa of Fabricius or kidney samples of the virulent strain AH726 with 80% mortality were 1.7–5.4 folds less than the levels of strain HN, respectively (*p* < 0.05).

## Discussion

Since 2015, a highly contagious disease with severe hydropericardium syndrome- inclusion body hepatitis (HPS-IBH) has spread in large-scale broiler farms in China [6, 17]. HPS-IBH disease results in a huge economic loss through caused a large number of chicken’s death [16]. In our study, five hypervirulent strains were isolated from several chicken farms in Eastern China and characterized to be serotype 4, based on phylogenetic analysis of the full length of genomic sequences. The whole genome sequences of five highly pathogenic FAdV-4 isolates have enhanced the current understanding of the pathogenesis of FAdVs. Meanwhile, the size of five FAdV-4 isolates in China was significantly different from that reported previously [18]. Alignment of the nucleotide sequence revealed a deletion in ORF19 and ORF29 (Fig. 2). However, the effects of the deletion and sequence differences in ORF19 and ORF29 on viral replication and pathogenicity remain to be studied.

To characterize the virulence of the new isolates, the purified strains were used to challenge SPF chickens and were found to cause 80% (strains AH726 and JS07) to 100% (strains HN, AQ and AH712) mortality. Dead chickens showed severe HPS-IBH, which was almost identical to those of naturally infected chickens (Figs.3, 4 and 5). The fact that these strains contain a truncated *ORF29* gene and are highly pathogenic suggests that the deletion of *ORF29* gene might influence the virulence of the novel Chinese FAdV-4 isolates.

Comparison with all the genomes in Genbank database showed minor differences in the pTP, 52 K, and 100 K proteins (Fig. 2). Cytokine gene expression associated with T helper 1 (Th1), pro-inflammatory, and immune-regulatory activities, namely IL-1β, IFN-α, IFN-β, IFN-γ, and IL-10, were investigated in the liver, bursa, and kidney (Fig. 6). The mRNA expression levels of proinflammatory cytokines (IL-6 and IL-8) and interferon-stimulated genes (Mx and OAS) were significantly upregulated by a recent virulent isolate SD0828 in chickens and tissue cultures [19]. However, most of the cytokines showed slight upregulation in the chickens without remarkable changes. The FAdV-4 avirulent strain ON1 (accession No. GU188428) induced neutralizing antibodies as well as the expression of cytokines, such as IFN-γ and IL-10 in the liver [20]. In the current study, cytokine expression was monitored following FAdV-4 infection (Fig. 5) and a remarkable increase in pro-inflammatory cytokine expression was observed. IFN-α, IFN-β, and IFN-γ are critical for host defense against a variety of pathogens [20–22]. IL-10 is a pleiotropic cytokine that has both immunosuppressive and immunostimulatory effects on many cell types [20]. Studies have shown in chickens and mammals IL-10 can inhibits Th1 responses [23], thus the T cell activity is inhibited. In the current study, IL-10 in the liver was significantly up-regulated at 3 d.p.i. in the infected group. Our finding is in agreement with previous reports [20], and may indicate an immune evasion mechanism used by the virus to support persistence.

In summary, the recently outbreak of HPS-IBH in eastern China was associated with a new type of hypervirulent FAdV serotype 4 variant, and the mortality was much higher than previously reported mortality. The chicken infection model established in this study will be important for the evaluation of antiviral therapy for FADV infection.

## Conclusions

These data will help us to better understand the molecular epidemiology and genetic diversity of the currently circulating FAdV-4 strains in Eastern China. Meanwhile, and provide new ideas for studying the molecular mechanism of FAdV-4 pathogenicity and immune evasion.

## Methods

### Sampling and virus amplification in specific pathogen free (SPF) embryonated eggs

In this study, chicken liver samples were collected and isolated from a San-Huang chicken layer farm with IBH and HPS in three provinces (Jiangsu, Shandong, and Anhui) between December 2015 and December 2016. Then, the liver samples were homogenized in 10% (wt/vol) in 0.01 M phosphate-buffered saline (pH 7.4) containing an antibiotic-antimycotic solution (Gibco BRL, Grand Island, NY). The homogenates were centrifuged at 10,000×g for 10 min after three freeze–thaw cycles. The supernatants were passed through 0.45-μm filters. The homogenates were inoculated into 7-day embryonated chicken eggs and then the embryonated eggs were incubated for 7 days, which were obtained from Merialvital Co. (Beijing, China). The allantoic fluids were collected in vials. The viruses were purified by end-point dilution for three times in SPF eggs. The viral titers were determined and calculated according to the Reed-Muench method for 50% egg lethal dose (ELD_50_) following the protocol from a previous study [24].

### Purification in CEK cells by plaque assay

Viruses were amplified and purified in CEK cells [25]. Briefly, primary CEK cells were prepared from the kidneys of 16–18 days-old SPF chicken embryos (SPF chicken embryos were obtained from Beijing Merial Vital Laboratory Animal Technology Co., Ltd., Beijing, China) and maintained in Dulbecco’s Modified Eagle Medium (DMEM) (Gibco) supplemented with 10% fetal bovine serum. The allantoic fluids containing the isolates were used to infect CEK cells at a confluency of 90% and the culture medium was replaced with DMEM supplemented with 2% fetal bovine serum (Gibco). The infected CEK cells were maintained at 37 °C and 5% CO_2_, and the supernatants were harvested at 72 h post-inoculation (hpi) when characteristic cytopathic effects (CPE) were observed. The isolates were then purified three times by plaque assay and subsequently amplified in CEK cells. The stock virus of each generation was stored at − 80 °C for titration.

### Determination of 50% tissue culture infection doses (TCID_50_)

The TCID_50_ values of the isolates were determined using 96-well plates (Thermo Scientific, USA) coated with Leghorn male hepatoma chicken liver (LMH) cells (ATCC CRL-2117). LMH cells were infected with tenfold dilutions of virus stocks from 10^− 1^ to 10^− 10^ in triplicate for each dilution and three wells for the negative control. The plates were incubated at 37 °C and plaques were observed by microscope on a daily basis. After 5 days of incubation, the TCID_50_ value was determined according to the Reed and Muench method [24].

### Polymerase chain reaction (PCR) and genome sequencing

The isolates were identified as FAdV-positive by PCR with primers *hexon-852F* and *hexon-1518R*. The primers, highly conserved among all the FAdV isolates, were used to amplify a 667-bp fragment in the *hexon* gene (from position 852 to 1518 based on the *hexon* gene of FAdV-4 strain ON1). For full-length sequencing, primers were designed. Sequences are shown in Additional file 1: Table S1. All the PCR products were generated with the following protocol: 94 °C for 5 min followed by 30 cycles of 94 °C for 30 s, 55 °C for 1 min, 72 °C for 3 min and then 72 °C for 10 min. The PCR products were gel-extracted and sequenced using Sanger method by Genewiz (Suzhou, China). The nucleotide sequences of the FAdV isolates were assembled and aligned with homologous sequences using the Lasergene 10 sequence analysis software package (DNAStar, Madison, WI). The phylogenetic tree for the complete genomes was constructed by MEGA6 using a maximum-likelihood method with 1000 bootstrap replicates. The schematic representations of the complete genome sequences were drawn by SnapGene software (GSL Biotech, Chicago, IL).

### Transmission electron microscopy

The primary CEK cells were seeded on coverslips and infected with 10 m.o.i. strain HN virus for 48 h, then the supernatants were removed and the cells were washed thrice with PBS. The infected CEK cells were then scraped and centrifuged in PBS buffer, discard the supernatants and fixed in phosphate buffered 2.5% glutaraldehyde buffer for 1 h. The virus pellets were then negatively stained with 1% phosphotungstic acid (Sigma, USA) for 30 s and mounted onto one-slot formvar nickel grids. The samples on grids were observed by transmission electron microscope (Tecnai G2 Spirit BIOTWIN, Netherlands).

### Pathogenesis in SPF chickens

To determine the pathogenicity of the isolates, animal experiments were performed using three-week-old SPF White Leghorn chickens (9 chickens/group, obtained from Merial Vital Laboratory Animal Technologies Co., LTD, Beijing, China.) challenged intramuscularly (I. M) with the isolates at a dose of 1 × 10^6^ TCID_50_/100 μl. The negative control was challenged with 100 μl sterile PBS. At 1, 2, and 3 days post-challenge (dpc), the oralpharyngeal and cloacal swabs were collected and determined for virus shedding. After 3 dpc, three chickens in each group were euthanized using carbon dioxide, and organs were collected and fixed in either 10% neutral formalin buffer (pH 7.2) or at − 80 °C for RNA extraction. The survival of the remaining chickens was monitored for 14 days and then euthanized by carbon dioxide.

### Quantification of viral load in tissues

Total DNA was extracted with DNeasy® tissue kit (Qiagen Inc., Gaithersburg, MD) and the viral load in the tissues was determined using a TaqMan probe fluorescence quantitative polymerase chain reaction (qPCR) method. The FAdV-4 *hexon* gene was used as an indicator for the presence of viral DNA. The forward and reverse primers were *hexon-1293F* and *hexon-1417R,* respectively. The TaqMan probe was hexon-Probe (Additional file 1: Table S2). To generate the FAdV-4C DNA standard curve, the target sequence located between 1293 nt to 1417 nt of *hexon* gene was cloned into the pGEM-T easy vector to create a pGEM-T-hexon plasmid. Copy numbers of the viral DNAs in the selected tissue specimens were calculated by comparison of the standard curve based on the positive template of the pGEM-T-hexon plasmid as previously described [26, 27].

### Real-time RT-PCR for quantification of cytokine mRNA levels

Real-time qRT-PCR assay for tissues was used as previously described [28, 29]. Briefly, approximately 100 mg of tissues were diluted with 1 mL sterilized PBS then homogenized at a speed of 7000×g for 2 min by TissueLyser-PEII apparatus (Jingxin, Shanghai, China). After centrifugation at 10,000×g for 5 min, 140 μL aliquots of the supernatants were collected for RNA extraction with RNeasy Mini kit (Qiagen), then the mRNA was used for cDNA synthesis with Oligo (dT)18 (Promega Co., Madison, WS, USA). Primers of the *β-actin* and pro-inflammatory cytokine genes (Additional file 1: Table S2) were used for real-time qRT-PCR analysis with AceQ® qPCR SYBR® Green Master Mix (Vazyme Inc., Nanjing, China) according to the following cycle protocol: 95 °C for 5 min, 40 cycles at 95 °C for 10 s (s) and 60 °C for 30 s, or followed by the melt curve stage: 95 °C for 15 s, 60 °C for 1 min, and 95 °C for 15 s.

### Immunohistochemistry assay

Tissues were fixed in 10% neutral formalin buffer, dehydrated, embedded, and then sectioned for Hematoxylin & Eosin (H & E) staining. The immunohistochemistry (IHC) assay of the liver and kidney slides with typical lesions was carried out as previously described [30]. Briefly, the IHC analysis was performed with monoclonal antibody (mAb) 1B4 specific for the detection of the Hexon protein using the avidin-biotin-peroxidase, which was prepared in the lab. Tissue sections were incubated with the primary mAb 1B4 (1:1, 000) at 4 °C for 6 h and next blocked with 2% BSA for 1 h and then incubated with the biotinylated secondary antibody and avidin-biotin-peroxidase complex (Boster, Wuhan, China) (1:2, 000). Representative images were caught under a Nikon microscope with an Olympus DP25 camera.

### Statistical analysis

Results are presented as means ± standard errors of the mean. All the data were graphed and statistical analyses were performed using Prism 7 software (GraphPad, La Jolla, CA). Two groups’ means were compared with a paired two-tailed student *t* test, whereas multiple comparisons were carried out using analysis of variance (one-way ANOVA method). The differences were considered statistically significant at *p* values of < 0.01 or < 0.05.

## Supplementary information

**Additional file 1: Table S1**. Primers used to amplify the complete genomic sequence of FAdV-C4v. **Table S2**. Primers for PCR identification and quantitation by Real-time PCR. **Table S3**. Comparison of variable amino acid sequences from Fiber-2 among HPS and non-HPS isolates.

## Data Availability

The datasets used and/or analyzed during the current study are available from the corresponding author on reasonable request.

## Abbreviations

bp: base pairs
CEK: Primary chicken embryo kidney
CPE: Cytopathic effects
Ct: Threshold cycle
dpc: day post-challenge
ELD_50_: 50% egg lethal dose
FAdV-4: FAdV serotype 4
H & E: Hematoxylin & Eosin
hpi: hours’ post-inoculation
HPS: Hydropericardium syndrome
IBH: Inclusion body hepatitis
IHC: Immunohistochemistry
LMH: Leghorn male hepatoma chicken liver
mAb: monoclonal antibody
OsO_4_: Osmium tetroxide
PBS: Phosphate-buffered saline
PCR: Polymerase chain reaction
RT: Reverse transcription
TCID_50_: 50% tissue culture infection doses
Th1: T helper 1 cells
