# Supplementary material for: Characterization and pathogenicity of fowl adenovirus serotype 4 isolated from eastern China
Source: BMC Vet Res. 2019 Oct 28;15:373. doi: 10.1186/s12917-019-2092-5 (PMC6816224; doi:10.1186/s12917-019-2092-5)
Supplement: Supplementary file 1 — Additional file 1: Table S1. Primers used to amplify the complete genomic sequence of FAdV-C4v. Table S2. Primers for PCR identification and quantitation by Real-time PCR. Table S3. Comparison of variable amino acid sequences from Fiber-2 among HPS and non-HPS isolates. [file 12917_2019_2092_MOESM1_ESM.docx]

**Table.S1 Primers used to amplify the complete genomic sequence of FAdV-C4v**

| **Primer pairs** | **Location, bp** | **upstream primer** | **Downstream primer** |
| --- | --- | --- | --- |
| 1 | 1~1213 | CATCATCTTATATAACCGCGTCT | CCTCCTCGGATCGTGTCAT |
| 2 | 1150~2399 | TTGAGCGAATCTTTACACCG | TCCACCATAGTTCCCTCCC |
| 3 | 2263~3620 | GTGGATTGGCGGAATAGGG | TTGGGTTGACGAAGTAAGAGCA |
| 4 | 3385~4736 | GCGTCCTTCTTGATCCTCG | CGTCCACCTGTCCTGCTTC |
| 5 | 4604~5978 | ACTTGATCGTCTTCGGGTGTC | CCTGTTCCTCCAACTGCCTC |
| 6 | 5858~7294 | GGATGCTACTCTGGCGTTGT | CGACTCCTTTCGCTGGTG |
| 7 | 7113~8510 | GCGAGTCTGAGGGAGAAATG | CCACAACGAGCAGCTAACG |
| 8 | 8414~9780 | GGGGTGTTCGGTGTCGTA | TAGACATCATCACGCTTCACAA |
| 9 | 9592~10994 | GGGCGTTGCTGAGCATTT | CACCTTACCGTCCGATTTCTA |
| 10 | 10704~12234 | ATGAATCGCACAAAGACGG | AGACAAGTCGGGAGACATCG |
| 11 | 12038~12947 | GATGGTATCGCTGTTGGAAGTC | GTCACCGACAGATCCGGATTAC |
| 12 | 12043~13410 | TATCGCTGTTGGAAGTCGC | AGAGGAGTCGTCGTGGGTC |
| 13 | 13173~14679 | TCTGTACGTGCTTTCGGTGGT | GCTGCGGGTTCAGTTTGA |
| 14 | 14382~15858 | TATCGCTCGGGACAGGTAGT | GCCGTAGTCGTAGAAGGTGC |
| 15 | 15616~16953 | TTGCTCCGCTTGTTCGTG | CGGTAAGTGTCCCTTAATAATGG |
| 16 | 16835~18130 | GCGGAATCAGAGGGTCGGGACT | ATCGGGCACCGTCAGCAAGG |
| 17 | 17973~21930 | AACGCTGCTCCCCTTTTA | GCCCGTAGTCAGGTCTCG |
| 18 | 18919~20333 | ACAGACAGGACGGACCAGC | TGCGAACCTAGACGAAACG |
| 19 | 20097~21857 | GAGATGGTGACGGAGGTG | CCAGTTTCTGTGGTGGTTG |
| 20 | 21568~23237 | CCAACGCCACTACCAACT | GAAAGCGGTGACGAGGAT |
| 21 | 22907~24378 | GTGGACCATCCCGTTCAGT | GCATCGAGCAGTGCGTGT |
| 22 | 24199~25468 | TGTGCGGGTGCTTGTGGT | GCGAGGTAGGAGGCGACTAA |
| 23 | 25107~26540 | CTGGTCGTCTTCTTCTTCGG | CAGAGTCGCTAGAGTGGCTAAA |
| 24 | 26360~27615 | CGGTTACTATTCGGCAGATGG | GATAAGCCTCGATGGTTTCCT |
| 25 | 27370~28699 | CCTTCCATCACGGTTTCG | TGCTCATCTGGTCCTCTTCC |
| 26 | 28608~29818 | GCCCGAAATCTACAATCCC | ACCTCCCATCATGCCTCC |
| 27 | 29671~30936 | CAGACCAACAGCCCTACGC | CGAGCACTTTGAGCACCC |
| 28 | 30816~32220 | GCCACTAAGCAAGTCAACG | CCTGATCCACGAGCAAGGT |
| 29 | 32096~33214 | ACGATGACTGGGAACTGGC | GGACAAATGGACGATCAATAAA |
| 30 | 33018~34357 | CCGCTACACCCTTCTATGCT | CGGTCCCTTCTGTGATTGC |
| 31 | 34213~37633 | CGGAGATTTGCGATTGTGAGT | TGACTCATCATGGGTGTGGC |
| 32 | 37492~38992 | ACACTAACTTCCTCATTGACCCGC | TGTCTGTCTGAACCTGCCTACC |
| 33 | 38879~40050 | ACGATGGCGTGATAGGCGGAGC | ATGAACCGTAGCCCCGCCCTTT |
| 34 | 39662~30916 | ACTACCGAGATCAGCCTGAAGA | CAGACTAAGGGAAAGTTGGAGAA |
| 35 | 40745~42991 | GAAATGCTTCCTCCTTCACG | TAGTGCCTGTCCATTTGCC |
| 36 | 42744~44196 | CTCACCAAAGCCCGTCTG | TTGATTCGGTGGAGGTCGT |
| 37 | 43995~45309 | CCCACTACCACTACCACCAC | ATCACGCTGACGCTCCTCC |
| 38 | 44722~45667 | AGCATGAATCAACTCGGTGTC | CATCATCTTATATAACCGCGTCT |

**Table.S2 Primers for PCR identification and quantitation by Real-time PCR**

| Name | Sequences (5'-3') | Size (bp) | Reference |
| --- | --- | --- | --- |
| *FAdV4-852F* | CAACTACATCGGGTTCAGGGATAACTTC | 667 | This study |
| *FAdV4-1518R* | CCAGTTTCTGTGGTGGTTGAAGGGGTT |  |  |
| *hexon-1293F* | CCAGAGGCGCAACTTTAT | 95 | This study |
| *hexon-Probe* | FAM-TGCCCGACCGTTACAAGTTTAGCA-BHQ1 |  |  |
| *hexon-1387R* | ATGTACTCGTAGGTGGTAGG |  |  |
| *qckIL1b-339F* | CTCACAGTCCTTCGACATCTTC | 117 |  |
| *qckIL1b-457R* | TGTTGAGCCTCACTTTCTGG |  |  |
| *qckIL10-432F* | TGCGAGAAGAGGAGCAAAG | 106 |  |
| *qckIL10-537R* | AGCAGGTACTCCTCGATGTA |  |  |
| *qckIFNα-613F* | CGTTATAGCCTGCCACTACTT | 121 |  |
| *qckIFNα-733R* | CCGTGGCGTCTGATCTTAAT |  |  |
| *qckIFNβ-333F* | CCTCAACCAGATCCAGCATTAC | 140 |  |
| *qckIFNβ-472R* | GCTGTAGGAAGTTGTGGATGG |  |  |
| *qckIFNγ-367F* | CTCCCGATGAACGACTTGAG | 111 |  |
| *qckIFNγ-477R* | CTGAGACTGGCTCCTTTTCC |  |  |
| *qβactin-771F* | TCCCTGGAGAAGAGCTATGAA | 113 |  |
| *qβactin-883R* | CAGGACTCCATACCCAAGAAAG |  |  |

**Table.S3 Comparison of variable amino acid sequences from Fiber-2 among HPS and non-HPS isolates**

|  | Isolate | Amino acid position | | | | | | | | | | | | | | | | | |
| --- | --- | --- | --- | --- | --- | --- | --- | --- | --- | --- | --- | --- | --- | --- | --- | --- | --- | --- | --- |
|  |  | 11-15 | 219 | 232 | 261 | 300 | 305 | 306 | 307 | 319 | 329 | 378 | 380 | 400 | 435 | 439 | 453 | 459 | 478 |
| HPS | HN | ENGKP | D | Q | T | T | A | N | A | I | L | T | T | G | S | E | A | N | L |
|  | AQ | ENGKP | D | Q | T | T | A | N | A | I | L | T | T | G | S | E | A | N | L |
|  | JS07 | ENGKP | D | Q | T | T | A | N | A | I | L | T | T | G | S | E | A | N | L |
|  | AH712 | ENGKP | D | Q | T | T | A | N | A | I | L | T | T | G | S | E | A | N | L |
|  | AH726 | ENGKP | D | Q | T | T | A | N | A | I | L | T | T | G | S | E | A | N | L |
|  | HB1510 | ENGKP | D | Q | T | T | A | N | A | I | L | T | T | G | S | E | A | N | L |
|  | JSJ13 | ENGKP | D | Q | T | T | A | N | A | V | L | T | T | G | S | E | A | N | L |
|  | MX-SHP95 | - | D | Q | N | T | A | H | A | V | L | T | T | A | S | D | A | A | V |
| Non-HPS | ON1 | - | G | E | S | I | S | H | P | V | V | A | A | A | T | D | S | A | V |
|  | KP5 | ENGQP | G | E | S | I | S | H | P | V | V | A | A | A | T | D | S | A | V |
